# Relationship between Depressive Symptoms, Personality, and Binge Drinking among University Students in Spain

**DOI:** 10.3390/jcm11010053

**Published:** 2021-12-23

**Authors:** Manuel Herrero-Montes, Cristina Alonso-Blanco, María Paz-Zulueta, Amada Pellico-López, Laura Ruiz-Azcona, Carmen Sarabia-Cobo, Víctor Fradejas-Sastre, Ester Boixadera-Planas, Paula Parás-Bravo

**Affiliations:** 1 Department of Nursing, Faculty of Nursing, University of Cantabria, 39008 Cantabria, Spain; manuel.herrero@unican.es (M.H.-M.); amada.pellico@scsalud.es (A.P.-L.); laura.ruiz@unican.es (L.R.-A.); carmen.sarabia@unican.es (C.S.-C.); victor.fradejas@unican.es (V.F.-S.); paula.paras@unican.es (P.P.-B.); 2 IDIVAL, Research Nursing Group, 39008 Cantabria, Spain; 3 Department of Physiotherapy, Occupational Therapy, Rehabilitation, and Physical Medicine, University Rey Juan Carlos, 28922 Madrid, Spain; cristina.alonso@urjc.es; 4 IDIVAL, GI Derecho Sanitario y Bioética, GRIDES, 39008 Cantabria, Spain; 5 Cantabria Health Service, 39340 Cantabria, Spain; 6 Servei d’Estadística Aplicada, Universitat Autònoma de Barcelona, 08193 Cerdanyola, Spain; ester.boixadera@uab.cat

**Keywords:** binge drinking, alcohol-related disorders, young adulthood, alcohol drinking in college, personality, depressive symptoms

## Abstract

Binge drinking (BD) is a common practice among college students. Alcohol consumption has been related to depressive symptoms and certain personality factors, although less is known about the relationship of these variables with BD. The aim of this study was to analyze the relationship of BD with depressive symptoms and personality in university students. We performed a cross-sectional study among students (aged 18–30 years) enrolled in the academic year 2018–2019 at the Faculty of Nursing of the University of Cantabria (Spain). Sociodemographic, academic, and alcohol and other drug use information was collected by means of a semi-structured questionnaire. The Beck Depression Inventory-II was used to measure depressive symptomatology and the Neo Five-Factor Inventory was used for personality. A total of 142 participants were included, 88.03% of which were women. Up to 38.03% were classified as BD. Concerning depressive symptoms, 7.41% of BD were at the moderate-severe level compared to 3.41% of non-BD (*p* = 0.7096). Regarding personality, the median score for extraversion of BD was 35 (Q1 = 29, Q3 = 40), 32 (Q1 = 28, Q3 = 36) in non-BD (*p* = 0.0062), conscientiousness scored 34 (Q1 = 30.5, Q3 = 38) points in non-BD, 31.50 (Q1 = 27, Q3 = 37) in BD (*p* = 0.0224). In conclusion, BD students have higher levels of extraversion and lower levels of responsibility than non-BD students. No significant differences were found between the level of depressive symptomatology between BD and non-BD students.

## 1. Introduction

In many societies, drinking alcoholic beverages is a common habit. It is estimated that more than 40% of the world’s population, around 50% in Europe, have consumed alcohol in the past year [1]. The prevalence of alcohol consumption is higher in Spain, where 90% of the population has consumed alcohol at some time in their lives and almost 8 out of 10 individuals have consumed alcohol in the last year [2]. Young adults are frequent consumers of alcohol [3]. Worldwide, it is estimated that 1 in 4 young people between 15 and 19 years of age are alcohol consumers, and this habit is especially prevalent in the WHO European Region. In Spain, approximately 60% of people between 15 and 34 years of age have consumed alcohol in the past 30 days [2].

Binge drinking (BD) is a pattern of alcohol consumption that consists of a high intake in a short period of time [4]. In 2016 the prevalence of this pattern worldwide among those aged 15 years and older was 18.2%, with the European region having the highest rates at 26.4% [1] and associated mainly with the male gender [2,5]. In Spain, the prevalence of BD is slightly lower compared to global rates, shown by approximately 15% in the general population. This pattern of consumption is especially frequent among young people, for example, approximately 30% of men between 20–29 years of age and around 20% of women of the same age indulging in BD [2].

Alcohol consumption and BD are behaviors especially linked to university life [6,7,8]. A prevalence of BD above 40% has been found in the last 30 days among university students [6,7,9], higher than those of young people of the same age who are not exclusively university students [10].

Depression is a mental health problem characterized by loss of interest in and enjoyment of ordinary things and experiences, subdued mood, and a range of associated emotional, cognitive, physical, and behavioral symptoms [11]. Depressive symptomatology refers to the set of signs and symptoms associated with depression. A systematic review conducted in 2011 showed that there is a moderately strong association between alcohol use disorders and major depression, even suggesting that there is a causal relationship, such that higher alcohol consumption increases the risk of depressive disorder [12]. In another study, a positive association was also found between the frequency of BD episodes with depressive symptoms from adolescence through young adulthood, and these associations were significantly stronger in females than in males [13].

Personality can be defined as “a person’s dynamic, internal organization of psychological systems that create characteristic patterns of a person’s behavior, thoughts and feelings” [14]. The Five Factor or Big Five model, belonging to the personality trait perspective, has become the dominant paradigm for research in the field of personality [14,15]. The five factors, using the terminology proposed by Costa and McCrae, are as follows [16]: Openness, Conscientiousness, Extraversion, Agreeableness, and Neuroticism. Relationships have been established between different personality traits and alcohol consumption: Malouff et al. [17] in a meta-analysis related alcohol consumption with high values in neuroticism and low values in agreeableness and conscientiousness. This same personality profile is found in patients who smoke. When BD practice is studied specifically, extraversion is the factor that is most strongly related to BD, as well as to the frequency of this practice and its negative consequences; conscientiousness is negatively related to BD, although samples with high values have been found, especially in men; neuroticism is positively related to BD practice; and neuroticism is positively related to BD [18].

The main objective of this work was to deepen the study of the association between depressive symptomatology and personality and BD alcohol consumption pattern in university nursing students in Spain.

## 2. Materials and Methods

### 2.1. Study Design

A cross-sectional study was performed. The study design was approved by the Ethics Committee of Cantabria, Spain (Code: 2015.102). All procedures were conducted according to the Declaration of Helsinki [19] and participants read and signed a written consent form prior to their participation in the study. The data were anonymized and treated confidentially in accordance with the Personal Data Protection legislation [20].

### 2.2. Participants

**Figure 1:**
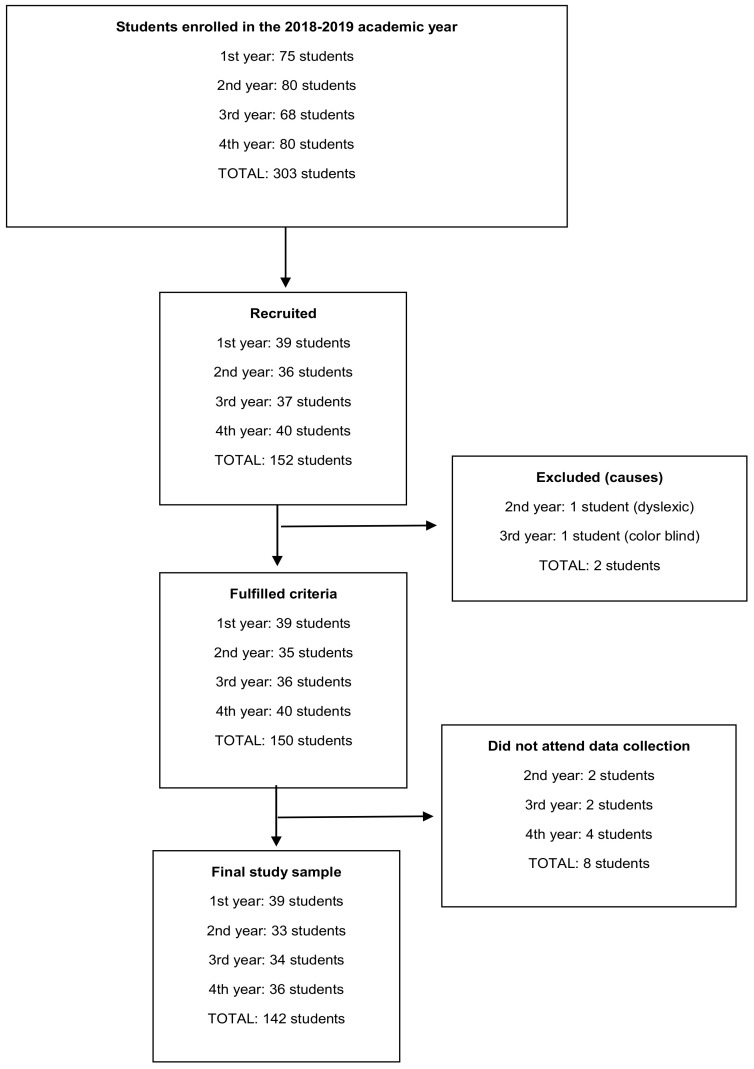
Diagram of study participation.

All students enrolled in the 2018–2019 academic year at the Faculty of Nursing of a University in the northern Spain aged between 18 and 30 years were included in the study. Those with sensory deficits or diseases that prevented the performance of the neuropsychological tests were excluded. Of 303 students enrolled, 142 finally participated in the study (Figure 1).

### 2.3. Variables and Measurement Tools

#### 2.3.1. Sociodemographic, Academic, and Alcohol and Other Drug Consumption Variables

A semi-structured interview was conducted where sociodemographic and academic variables were collected (gender, age, place of residence, mother and father’s level of studies, and average grade on the academic transcript) and questions related to the use of alcohol (volume, speed, and frequency of consumption; age of onset of alcohol consumption; etc.). The questionnaire included the Alcohol Use Disorders Identification Test (AUDIT) developed by the WHO [21] which has been validated in Spain by Rubio et al. [22] and that has adequate psychometric properties in the detection of alcohol problems in college students [23], as well as for the distinction between BD and non-BD consumption in this population group [24]. In addition, data on tobacco, cannabis or cocaine use in the last year were collected by asking: “During the last 12 months, how often have you used tobacco/cannabis/cocaine?”.

From the data obtained, students were classified as BD or non-BD according to the definition proposed by Parada et al. [25]: “consumption of 6 or more alcoholic drinks for men (60 g)-5 or more for women (50 g)-on a single occasion (within a 2-h period) at least once in the past 30 days”.

#### 2.3.2. Depressive Symptoms

The Beck Depression Inventory-II (BDI-II) was used to measure depressive symptoms. This questionnaire is used to measure depressive symptoms in the general population, as well as in patients with psychological disorders [26]. This is a self-administered instrument with 21 items. Each of them offers four answers from least to most severe, except for items 16 and 18, which offer seven possibilities. The subject must choose the response that best suits his or her situation during the last two weeks. Each of the item’s scores between 0 and 3, and the score obtained in each response is added directly. The total score of the test varies between 0 and 63. The original manual of this test proposes four levels of depressive symptomatology (minimal, mild, moderate, and severe) depending on the total score obtained [27]. These cut-off scores are also considered adequate for Spanish university students [28].

The BDI-II has acceptable psychometric properties to assess depressive symptoms in Spanish adults [26] and has also been validated in a university population in Spain [28].

#### 2.3.3. Personality

The Neo Five-Factor Inventory (NEO-FFI) was used to measure the different personality dimensions. The NEO-FFI is the reduced version of the Revised Neo Personality Inventory (NEO PI-R). It provides a measure of the personality dimensions proposed by the five-factor model. The Spanish version is composed of 60 items and can be used to obtain a global view of personality in less time (15 min) than the NEO-PI-R [16]. The 60 items are divided into five scales, corresponding to the five personality factors, composed of 12 items each. The response options to each item are distributed on a Likert-type scale: totally disagree; disagree; neutral; agree; totally agree. A score between 0 and 4 is assigned to each item and a total score is obtained for each factor composed of the 12 items that comprise it. Thus, the maximum score for each scale is 48 and the minimum score is 0. The Spanish version has normative data its psychometric properties have been analyzed, making it a good instrument for personality research [29].

### 2.4. Procedure

Participants were recruited through informative sessions and posters displayed at the faculty. Two researchers oversaw data collection. Both were trained by a psychologist, who was a member of the research team, on how to administer the psychological tests. Two independent offices with good lighting and temperature conditions were available for this task. Data collection was carried out between December 2018 and January 2020. The data collection time was approximately 30 min per participant. Participants were required not to take alcohol or any other drug on the day of the assessment.

The data from the first 30 participants were used as a pilot study, and after their analysis the research team met without substantial changes to either the questionnaires or the administration procedure.

### 2.5. Statistical Analysis

For the statistical analysis, a distinction was made between categorical and quantitative variables. A descriptive bivariate analysis of BD and the rest of the study variables was performed. Categorical variables are presented as counts and percentages of the total and of the BD and non-BD groups. These descriptive results have been completed with Pearson’s chi-squared test, likelihood ratio chi-squared test, or Fisher’s exact test as appropriate to contrast the independence between each variable and BD. Quantitative variables are presented with basic descriptive statistics (*n*, mean, median, SD, and quartiles) for the total and for the BD and non-BD groups. These results are complemented with the non-parametric Kruskall–Wallis test to contrast the distributions of the quantitative variable between non-BD and BD. In cases of normal distribution of the variable in each non-BD and BD group, the results of the *t*-test to compare means of independent groups are also presented: non-BD and BD.

Statistical analysis was performed using SAS v9.4 software (SAS Institute Inc, Cary, NC, USA) Statistical decisions were made with a significance level of 0.05.

## 3. Results

In total, 142 participants were included in the study. Eighty-eight participants belonged to the non-BD group and 54 to the BD group, corresponding to 61.97% and 38.03%, respectively, of the total sample. Of the total, 11 participants (7.75% of the total) were abstainers, meaning that they had not consumed alcohol in the last year, and six of these had never consumed alcohol (4.22% of the total).

### 3.1. Sociodemographic, Academic, Alcohol, and Other Drug Use Variables

Women constituted 88.03% of the participants. A higher proportion of women was found in the non-BD group: 92.05% women and 7.95% men, whereas in the BD group there were 81.48% women and 18.52% men, although this difference was not statistically significant (*p* = 0.0598). A higher percentage of non-BDs lived in the family home and their mother’s academic level was higher than that of the BD group, although the differences were not statistically significant (Table 1).

Regarding alcohol and other drug use data (Table 2), participants in the BD group started drinking alcohol at an earlier age, had a greater number of drinking disorders, drank more frequently, and used tobacco and cannabis at a higher rate than non-BDs.

### 3.2. Depressive Symptoms

In the non-BD group, the median score was 5 (Q1 = 2.5, Q3 = 9) points versus 7.5 (Q1 = 3, Q3 = 12) points for BD; the difference was not statistically significant (*p* = 0.2476).

When categorized by terciles, 29.63% of the BD and 18.18% of the non-BD were found to be in T1, the one with the highest scores. Likewise, when the score was categorized by level of depressive symptomatology, a higher percentage of BD experiencing a greater severity of symptoms is observed: 7.41% of BD in the moderate-severe level versus 3.41% of non-BD in that level (*p* = 0.7096) (Table 3).

### 3.3. Personality

Statistically significant differences were observed between the non-BD and BD groups in the extraversion factor, both when analyzing the direct scores (*p* = 0.0062) and when categorized by terciles (*p* = 0.0429) with the median value being higher in the BD, 35 (Q1 = 29, Q3 = 40) points than in the non-BD, 32 (Q1 = 28, Q3 = 36) points. For the conscientiousness factor, the median scores were higher in the non-BD group, who scored 34 (Q1 = 30.5, Q3 = 38) points compared to the BD group, 31.50 (Q1 = 27, Q3 = 37) points; this difference was statistically significant (*p* = 0.0224). For the neuroticism, openness, and agreeableness factors, no statistically significant differences were observed when both direct scores and categorization by terciles were analyzed (Table 4).

## 4. Discussion

The percentage of BD students suffering from depressive symptomatology is higher than that of non-BD students and of greater intensity in our sample, however, no statistically significant differences were found.

In our study, the relationship between BD and depressive symptoms could be affected because the sample is mostly female, whereas in male university students, BD has been positively associated with depression, in women this relationship is negative [30]. Tavolacci et al. [31] also used the BDI-II and failed to find differences in university students between no BD, occasional BD, and frequent BD in a cross-sectional study, although their data point to less depressive symptomatology with a greater frequency of BD. Along the same lines, in a longitudinal study among college students, Haardörfer et al. [32] found that those who had greater depressive symptoms were more likely to engage in BD over time. Paljärvi et al. [33] conducted a longitudinal study among the general population, with a five-year follow-up, and found that the practice of BD at the beginning of the study was related to depressive symptoms five years later; according to the authors, BD contributed independently to the appearance of depressive symptoms. In the same vein, Boden et al. [12] claim that there is a causal relationship between alcohol use disorders and major depression, such that increased alcohol consumption increases the risk of depression.

In general, although with heterogeneous results, the different studies performed generally point to a certain relationship between depressive symptoms and BD, although the meaning and effect of one variable over the other is unclear. One of the motivations for university students to engage in BD is that it reduces depressive symptoms [34], therefore BD, could be seen as a method to reduce these problems, which is why higher rates of BD are found among students with greater symptoms. However, it is also possible that some of these students consume alcohol as a consequence of depressive conditions and not the other way around [35,36]. Furthermore, stress seems to be related to depressive symptomatology in university students [37]. The measurement of stress level should be included in future research.

These results can guide interventions for early detection and prevention of alcohol consumption and intensify them in students with depressive symptoms.

Regarding personality, BD students have higher levels of extraversion and lower levels of conscientiousness than non-BD students. Our results partially coincide with those of Adan et al. [38] also conducted in Spanish university students, however, this previous study used the Alternative Five Factor Model to measure personality traits, where the BD group participants scored significantly higher than controls in the dimensions neuroticism-anxiety and impulsive sensation seeking, which partially correspond in the Big Five model to high scores in neuroticism and low scores in conscientiousness [14].

Ibáñez et al. [39] in a study with adolescents aged 14–16 years in Spain and using the Big Five model, also found that extraversion and low conscientiousness predicted higher weekend drinking, while low agreeableness was associated with daily drinking.

Adan et al. [18] in a systematic review associated BD in the general population, with high levels of neuroticism and extraversion and low levels of conscientiousness, with extraversion being the factor most strongly related to BD.

A review by Kuntsche et al. [40] in non-university populations of studies that have used the “Five Factors” model, as in our case, mostly reveals a relationship between BD and high scores in extraversion but low scores in neuroticism, agreeableness, conscientiousness, and openness to experience.

Young people who are more outgoing might be more likely to participate in social and leisure activities, some of which involve alcohol consumption, and low conscientiousness might be related to the inability to stop BD patterns of alcohol consumption despite the consequences it can entail.

The present results support the body of research suggesting that certain personality traits increase the risk of BD. It could be interesting to systematically evaluate the personality of the students in order to be able to target alcohol prevention actions especially to young people who present certain personality patterns.

Our study suffers from some limitations. First, the absence of an instrument that identifies individuals who practice BD, probably since there is no international definition of this pattern of consuming alcohol. Second, the participants in this study are nursing students, mostly women. Third, it is a cross-sectional study and the average age of the participants is low.

Notwithstanding these limitations, our study presents several points of strength. First, we have used a definition of BD that includes the aspects that are usually included in other definitions and that is adapted to the circumstances of our country. Second, this study provides information on a very specific group that has rarely been studied. Third, despite the low age of the sample and the design of the study, differences between the groups in terms of depressive symptomatology and especially in personality are already detected, which will guide future research in this field, where it would be interesting to continue research with longitudinal studies if the findings are confirmed and also to explore the direction of the relationships—i.e., whether depressive symptoms and certain personality factors are the cause of BD-type consumption or a consequence of it.

## 5. Conclusions

No differences were found between the level of depressive symptomatology between college students maintaining a BD pattern and those without in our sample. Students with a BD pattern of consumption have higher levels of extraversion, whereas non-BD students have higher levels of conscientiousness, and there are no differences in neuroticism, openness to experience, and agreeableness. It could be interesting to systematically evaluate the personalities of the students in order to be able to target alcohol prevention actions, especially with young people who present certain personality patterns.

## Figures and Tables

**Table 1:** Socio-demographic and academic data.

	Total (*n* = 142)		Binge Drinkers			
			No (*n* = 88)		Yes (*n* = 54)	
	Mean	SD	Mean	SD	Mean	SD
**Age**	20.73	2.67	20.77	2.77	20.67	2.53
			*p* = 0.9830 ^a^			
**Mean grade**	7.18	0.9	7.24	0.96	7.07	0.79
			*p* = 0.1753 ^a^			
	*n*	%	*n*	%	*n*	%
**Gender**						
Female	125	88.03%	81	92.05%	44	81.48%
Male	17	11.97%	7	7.95%	10	18.52%
			*p* = 0.0598 ^b^			
**Place of residence**						
Family home	113	79.58%	68	77.27%	45	83.33%
Not in the family home	29	20.42%	20	22.73%	9	16.67%
			*p* = 0.3845 ^b^			
**Maternal level of studies**						
University	43	30.28%	33	37.5%	10	18.52%
Secondary/vocational training	57	40.14%	32	36.36%	25	46.3%
Primary	36	25.35%	21	23.86%	15	27.78%
No studies	6	4.23%	2	2.27%	4	7.41%
			*p* = 0.0631 ^b^			
**Paternal level of studies**						
University	45	31.69%	27	30.68%	18	33.33%
Secondary/vocational training	55	38.78%	36	40.91%	19	35.19%
Primary	36	25.35%	22	25.00%	14	25.93%
No studies	6	4.23%	3	3.41%	3	5.56%
			*p* = 0.8671 ^a^			

^a^ Wilcoxon test; ^b^ Chi-squared test; SD—standard deviation.

**Table 2:** Alcohol and other drugs consumption data.

	Total (*n* = 142)				Binge Drinkers							
					No (*n* = 88)				Yes (*n* = 54)			
	Mean	SD	Range	Median	Mean	SD	Range	Median	Mean	SD	Range	Median
**Age of onset of alcohol use (*n* = 136)**	15.24	1.65	8–19	15	15.67	1.66	8–19	16	14.59	1.43	12–18	15
					*p* < 0.0001 ^a^							
**AUDIT total**	4.96	4.55	0–20	3	2.94	2.92	0–14	2	8.26	4.83	1–20	7
					*p* < 0.0001 ^a^							
**SUBSTANCE USE IN THE LAST 12 MONTHS**												
	*n*		%		*n*		%		*n*		%	
**Alcohol**												
Less than monthly	41		28.87%		39		44.32%		2		3.7%	
1 to 3 times a month	57		40.14%		38		43.18%		19		35.19%	
Once a week	17		11.97%		5		5.68%		12		22.22%	
2 or more times per week	27		19.01%		6		6.82%		21		38.89%	
					*p* < 0.0001 ^b^							
**Tobacco**												
Never	99		69.72%		71		80.68%		28		51.85%	
Some days	32		22.54%		15		17.05%		17		31.48%	
Every day	11		7.75%		2		2.27%		9		16.67%	
					*p* = 0.0003 ^b^							
**Cannabis**												
Never	110		77.46%		79		89.77%		31		57.41%	
Some days	32		22.54%		9		10.23%		23		42.59%	
					*p* < 0.0001 ^b^							
**Cocaine**												
Never	142		100.00%		88		100.00%		54		100.00%	

^a^ Wilcoxon test; ^b^ Chi-squared test; SD—standard deviation; AUDIT—Alcohol Use Disorders Identification Test.

**Table 3:** Depressive symptomatology.

	Total (*n* = 142)				Binge Drinkers							
					No (*n* = 88)				Yes (*n* = 54)			
	Mean	SD	Range	Median	Mean	SD	Range	Median	Mean	SD	Range	Median
**BDI-II Total score**	7.46	6.86	0–37	6.00	6.83	6.34	0–37	5.00	8.48	7.57	0–37	7.5
					*p =* 0.2476 ^b^							
	*n*		%		*n*		%		*n*		%	
**BDI-II tercile**												
BDI-II ≤3	46		32.39%		29		32.95%		17		31.48%	
3 < BDI-II ≤11	64		45.07%		43		48.86%		21		38.89%	
BDI-II >11	32		22.54%		16		18.18%		16		29.63%	
					*p =* 0.2583 ^a^							
**Level of depressive symptomatology**												
Minimum (0–13)	121		85.21%		77		87.50%		44		81.48%	
Mild (14–19)	14		9.86%		8		9.09%		6		11.11%	
Moderate (20–18)	5		3.52%		2		2.27%		3		5.56%	
Severe (29–63)	2		1.41%		1		1.14%		1		1.85%	
					*p =* 0.7096 ^a^							

^a^ Wilcoxon test; ^b^ Chi-squared test; BDI-II—Beck Depression Inventory-II; SD—standard deviation.

**Table 4:** Personality.

	Total (*n* = 142)				Binge Drinkers							
					No (*n* = 88)				Yes (*n* = 54)			
**TOTAL SCORES**												
	Mean	SD	Range	Median	Mean	SD	Range	Median	Mean	SD	Range	Median
**Neuroticism**	20.37	8.24	3–44	20.00	20.39	8.52	3–44	20.00	20.33	7.85	7–39	20.50
					Difference: 0.05 CI (−2.77−2.88) *p =* 0.9705 ^a^							
**Extraversion**	32.58	7.30	10–47	33.00	31.32	7.09	10–44	32.00	34.63	7.24	14–47	35.00
					*p =* 0.0062 ^b^							
**Openness**	30.25	6.98	10–47	30.00	29.86	6.21	15–44	29.50	30.89	8.10	10–47	32.00
					*p =* 0.4273 ^b^							
**Agreeableness**	31.31	5.88	16–45	31.00	31.56	5.99	16–45	31.00	30.91	5.72	19–43	31.50
					Difference: 0.65 CI (−1.36−2.66) *p =* 0.4601 ^a^							
**Conscientiousness**	33.05	6.73	10–55	33.00	34.19	6.41	10–55	34.00	31.19	6.87	16–44	31.50
					*p =* 0.0224 ^b^							
**TERCILE**												
	*n*		%		*n*		%		*n*		%	
**Neuroticism (NEU)**												
NEU ≤ 13	37		26.06%		24		27.27%		13		24.07%	
13 < NEU ≤ 26	70		49.30%		41		46.59%		29		53.70%	
NEU > 26	35		24.65%		23		26.14%		12		22.22%	
					*p =* 0.7108 ^c^							
**Extraversion (EXT)**												
EXT ≤ 28	42		29.58%		32		36.36%		10		18.52%	
28 < EXT ≤ 37	67		47.18%		40		45.45%		27		50.00%	
EXT > 37	33		23.24%		16		18.18%		17		31.48%	
					*p =* 0.0429 ^c^							
**Openness (OPE)**												
OPE ≤ 25	37		26.06%		21		23.86%		16		29.63%	
25 < OPE ≤ 35	73		51.41%		51		57.95%		22		40.74%	
OPE > 35	32		22.54%		16		18.18%		16		29.63%	
					*p =* 0.1164 ^c^							
**Agreeableness (AGR)**												
AGR ≤ 28	45		31.69%		24		27.27%		21		38.89%	
28 < AGR ≤ 36	69		48.59%		47		53.41%		22		40.74%	
AGR > 36	28		19.72%		17		19.32%		11		20.37%	
					*p =* 0.2796 ^c^							
**Conscientiousness (CON)**												
CON ≤ 29	41		28.87%		21		23.86%		20		37.04%	
29 < CON ≤ 37	69		48.59%		44		50.00%		25		46.30%	
CON > 37	32		22.54%		23		26.14%		9		16.67%	
					*p =* 0.1793 ^c^							

^a^ T Test; ^b^ Wilcoxon test; ^c^ Chi-squared test; Neo Five-Factor Inventory—NEO-FFI; SD—standard deviation; CI—confidence interval; NEU—neuroticism; EXT—extraversion; OPE—openness; AGR—agreeableness; CON—conscientiousness.

## Data Availability

The data presented in this study are available on request from the corresponding author.
